# Long-term consequences of an intensive care unit stay in older critically ill patients: design of a longitudinal study

**DOI:** 10.1186/1471-2318-11-52

**Published:** 2011-09-02

**Authors:** Marie-Madlen Jeitziner, Virpi Hantikainen, Antoinette Conca, Jan PH Hamers

**Affiliations:** 1 Department of Intensive Care Medicine, Inselspital/Bern University Hospital and University of Bern, Bern, Switzerland; 2 Institute of Applied Nursing Science, University of St. Gallen, Switzerland; 3 Cantonal Hospital Aarau, Aarau, Switzerland; 4 Maastricht University, School for Public Health and Primary Care, Department of Health Care and Nursing Science, Maastricht, Netherlands

## Abstract

**Background:**

Modern methods in intensive care medicine often enable the survival of older critically ill patients. The short-term outcomes for patients treated in intensive care units (ICUs), such as survival to hospital discharge, are well documented. However, relatively little is known about subsequent long-term outcomes. Pain, anxiety and agitation are important stress factors for many critically ill patients. There are very few studies concerned with pain, anxiety and agitation and the consequences in older critically ill patients. The overall aim of this study is to identify how an ICU stay influences an older person's experiences later in life. More specific, this study has the following objectives: (1) to explore the relationship between pain, anxiety and agitation during ICU stays and experiences of the same symptoms in later life; and (2) to explore the associations between pain, anxiety and agitation experienced during ICU stays and their effect on subsequent health-related quality of life, use of the health care system (readmissions, doctor visits, rehabilitation, medication use), living situation, and survival after discharge and at 6 and 12 months of follow-up.

**Methods/Design:**

A prospective, longitudinal study will be used for this study. A total of 150 older critically ill patients in the ICU will participate (ICU group). Pain, anxiety, agitation, morbidity, mortality, use of the health care system, and health-related quality of life will be measured at 3 intervals after a baseline assessment. Baseline measurements will be taken 48 hours after ICU admission and one week thereafter. Follow-up measurements will take place 6 months and 12 months after discharge from the ICU. To be able to interpret trends in scores on outcome variables in the ICU group, a comparison group of 150 participants, matched by age and gender, recruited from the Swiss population, will be interviewed at the same intervals as the ICU group.

**Discussion:**

Little research has focused on long term consequences after ICU admission in older critically ill patients. The present study is specifically focussing on long term consequences of stress factors experienced during ICU admission.

**Trial Registration:**

ISRCTN52754370

## Background

Modern methods in intensive care medicine often enable the survival of older critically ill patients. The short-term outcomes for patients treated in intensive care units (ICUs), such as survival to hospital discharge, are well documented. However, relatively little is known about subsequent long-term outcomes [1]. It is generally recognized that survival alone is not the only important outcome following an ICU stay. Various stress factors experienced in the ICU and the severity of illness or injury have long-term consequences [2-4]. Over the long term, discomfort experienced in the ICU and stressful memories of an ICU stay have been associated with the development of acute posttraumatic stress disorder-related syndromes, anxiety, depression, and impaired health-related quality of life (HR-QOL) [5-9]. For some patients these symptoms are chronic and cause lasting personality changes. Other studies focus on outcomes such as functional status, ability to live at home, influence on the social network, and the burden on the family, all of which can, at times, be more important than the length of survival [10-12]. Recent research has indicated that there may be significant cognitive and emotional dysfunction following critical illness [13-15]. Another study shows that survivors of critical illnesses have increased health care system needs. Continuing hospital care, and/or rehabilitation, community support services or other healthcare services after the ICU stay were needed. In addition, there was an increase in the use of medication and of doctor visits [16]. An increasing proportion of critically ill patients are aged (65 years and older). Older patients with severe injuries are at risk of poor outcome [17]. Mortality rates are almost 22% in older surgical patients [18], and yet age alone does not appear to be a reliable predictor of outcome after ICU admission [19,20]. Older patients express preferences for longer life under compromised health conditions more frequently than healthy persons [21]. Until now, few studies have examined long-term outcomes after ICU admissions in older critically ill patients [17,22,23]. Pain, anxiety and agitation are important stress factors for many critically ill patients, yet these symptoms are difficult to distinguish from one another. Pain, anxiety and agitation can have consequences for the health-related quality of life [5]. In addition, pain, anxiety, and agitation, and their consequences for long-term outcomes, have rarely been examined in older critically ill patients in the ICU [24-26]. In contrast to other studies [22,23], the present study examines the relationship between the ICU stay and post-hospital pain, anxiety and agitation in older critically ill patients, and addresses whether acute experiences in the ICU can cause more serious chronic conditions after discharge. This article describes a longitudinal study in which older critically ill patients are followed one year after their discharge from the hospital. Under investigation were their pain experiences, levels of anxiety and agitation, health-related quality of life, and use of the health care system, in order to detect relationships between these main outcomes and their ICU experiences.

### Study Aims

The overall aim of this study is to identify how an ICU stay influences an older person's experiences later in life. The study addresses the following research questions: (1) Does an ICU stay influence the pain, anxiety, and agitation experienced by older critically ill patients after discharge, and at 6- and 12-month follow-up? (2) Do the pain, anxiety and agitation experienced by older critically ill patients during an ICU stay affect their experiences of these symptoms after discharge, and at 6- and 12-month follow-up? (3) Does an ICU stay affect the health-related quality of life (HR-QOL) of older critically ill patients after discharge, and at 6-and 12-month follow-up? (4) What is the relation between the ICU stay and subsequent use of the health care system (readmissions, general practitioner visits, rehabilitation, length of hospital stay), medication use, living situation, and survival as experienced by older critically ill patients after discharge, and at 6- and 12-month follow-up?

## Methods/Design

### Design

**Figure 1:**
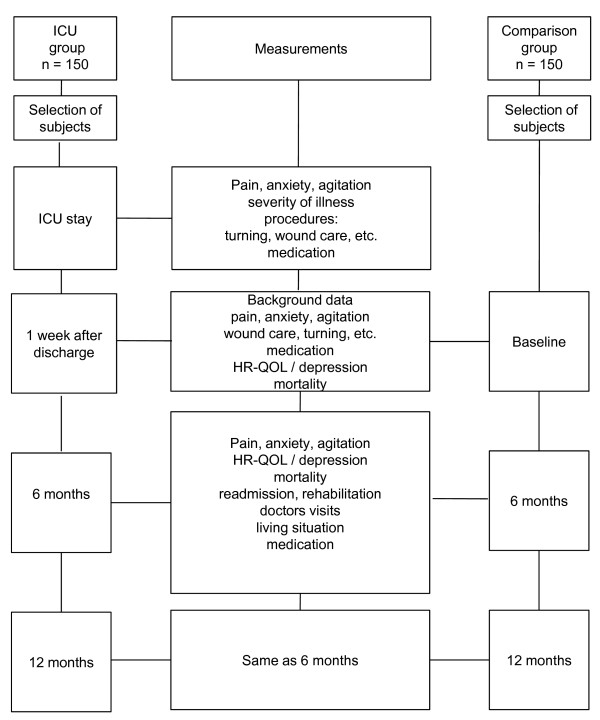
**Flow Chart of the Study Design**. Schedule of the Study and Measurements of the ICU and Comparison Groups.

A prospective longitudinal study will be conducted in older critically ill patients admitted to the ICU. Data will be collected over a period of two years at the following intervals: during ICU admission and 1 week, 6 months and 12 months after hospital discharge. A flow chart of the study is presented in Figure 1.

### Participants and Setting

The study will be conducted in the interdisciplinary ICU of a Swiss university hospital, where approximately 4000 patients with surgical procedures, illnesses, and trauma are treated each year, as well as at 12 follow-up clinics. From this ICU, 150 older critically ill patients (age 65 years and older) will be recruited, to be followed until 1 year after discharge from the hospital (the ICU group). A sample of 100 patients will be needed, based on a power analysis; with a 95% confidence interval for an estimated prevalence of pain between 0.30-0.50 and a total of 0.20, a sample size of 81-96 is needed. In order to ensure a large enough sample, and to counteract the potential loss of participants through mortality, relocation, attrition, or other problems, an additional 50 participants will be recruited (150 total). In order to be able to interpret trends in scores in the ICU group for the outcome variables *pain, anxiety*, *agitation, HRQOL*, *use of the health care system *(readmissions, GP visits, rehabilitation, length of hospital stay), *medication use, living situation*, and *survival*, we decided to collect similar data in a comparison group. Participants of the comparison group are a convenience sample of older people from the Swiss population, who have not been admitted to an ICU for the last 15 years. Through matching of age and gender, an attempt is made to increase the comparability of the groups at baseline.

### Inclusion and exclusion criteria

To participate in this study, the participants must have a minimum age of 65 years, have had an ICU stay of at least 48 hours (ICU group) or no stay for the last 15 years (comparison group), be able to speak and read either German or French, and live in Switzerland. Excluded were participants because of temporary tracheostomies, chronic mechanical ventilation, illness-related cognitive impairment (dementia), or psychotic illnesses including delusions and changes in mental state, and potentially terminal illnesses such as lung or heart diseases and cancer.

## Measurements

### Background data

Demographic data include age, gender and marital status. Furthermore, health-related data including Activities of Daily Living (ADL) and co-morbidities will be collected at baseline for all participants. The severity of the patient's condition will be determined using the Acute Physiology and Chronic Health Evaluation (APACHE II) and the Simplified Acute Physiology Score (SAPS II) [27]. The performance of ADL will be measured using items querying the need for assistance with daily activities (home health care, home food delivery, residence in a retirement home or nursing home) and various quality-of-life dimensions such as physical function, physical role function, vitality and social role function (Short Form 36 Health Survey [SF-36]). A scale developed for this study will be used to assess the need for assistance with daily activities (completely dependent; needs support by caregivers and special medical equipment; needs caregivers; independent with medical equipment; completely independent). Co-morbidities will be measured using the International Classification of Diseases (ICD), Version 10 [28].

### Pain, anxiety and agitation during an ICU stay

During the ICU stay, pain, anxiety and agitation will be measured in the ICU group. In patients unable to express pain themselves verbally, pain intensity will be measured using a behavioural pain scale employing three behavioural parameters--mimic, body movement, and muscle tone. The intensity of the behavioural parameters will be assessed on the basis of a four-point Likert scale: each characteristic will be rated as weak or absent, moderately severe, severe, or very severe. The validity of the scale has been determined to be adequate, and its interrater reliability is high (kappa value 0.80) (Jeitziner MM. et al. Assessment of pain in sedated and mechanically ventilated patients: an observational study, submitted). Furthermore, potentially painful procedures will be recorded. The painful procedures include: intratracheal suctioning, dressing changes, repositioning in bed, and insertion of a central venous catheter [29]. Anxiety will be assessed using a numeric rating scale (0-10) (0 = no anxiety to 10 = worst possible anxiety) [30]. Agitation during the ICU stay will be measured using the German version of the Richmond Agitation-Sedation Scale (RASS), which has 10 levels, ranging from -5: unarousable (no response) to +4: combative (danger to staff). This scale has proven reliable for the assessment of critically ill patients in the ICU, with strong interrater reliability and criterion, construct, and face validity [31,32]. The Confusion Assessment Method for the ICU (CAM-ICU) will be used to assess delirium in the ICU [33], as well as general cognitive abilities, focusing on the following factors: a) acute onset or fluctuating course, b) inattention, c) disorganized thinking, and d) altered level of consciousness. This scale has been proven reliable and valid, with an interrater reliability kappa value of 0.96. Its sensitivity was 100% and 93%, with 98% and 100% specificity [34].

### Outcome indicators after discharge

In line with the study aims, the following outcome indicators will be measured in both the ICU group and the comparison group: (1) pain, anxiety and agitation, (2) HRQOL and (3) use of the health care system (readmissions, general practitioner visits, rehabilitation, length of hospital stay), medication use, living situation and survival. Pain intensity will be measured with a numeric scale (NRS) (0 = no pain to 10 = worst possible pain) [29,30], and pain frequency with a Likert-type scale (never, seldom, occasionally, often, always). Anxiety will be assessed using a numeric rating scale (0-10) and the Hospital Anxiety and Depression Scale (HADS-D), which uses two subscales--an anxiety scale (HADS-A) and a depression scale (HADS-D). Internal consistency (Cronbach's alpha) is 0.80 for HADS-A and 0.81 for HADS-D. The test-retest reliability shows the correlations r = 0.81 for HADS-A and r = 0.89 for HADS-D [35]. Agitation will be measured using the Confusion Assessment Method (CAM). The German or French Short Form of the Confusion Assessment Method (CAM) [36], including a telephone version for the 6- and 12-month follow-ups [37], will be used for all participants. The inter-observer reliability of the CAM is high (kappa = 0.81-1.0) [38]. Health-related quality of life (HR-QOL) will be assessed using the component scores of the Short Form 36 Health Survey (SF-36). As a comprehensive, generic, 36-item instrument, the SF-36 concentrates on the subjective evaluation of health: 1) physical function, 2) physical role function, 3) bodily pain, 4) general health perceptions, 5) vitality, 6) social role function, 7) emotional role function, and 8) mental health. The items vary from yes/no questions to those offering 6 levels of choice. All subscales have been adapted to yield assessment values between 0 and 100. The health care system assessment will include the number, cause, and length of hospital admissions and stays using APACHE-Diagnosis non-operative and operative diagnoses (1) cardiovascular, 2) respiratory, 3) gastrointestinal, 4) neurological, 5) sepsis, 6) trauma, 7) metabolic, 8) haematological, 9) other, 10) cardiologic surgery, 11) respiratory surgery, etc., the number and the length of rehabilitation periods (location, length, ambulatory, stationary, discharge location) and the number of GP visits. Medication therapy, and mortality incidence, will be collected from all subjects in the ICU and comparison groups over the course of the study. Medication therapy includes all sedative, analgesic, antipsychotic, psychotropic, steroidal, and vasoactive medications. The cumulative amount of medication will be recorded. Mortality incidence will be determined based on survival rates. For all instruments, French and German versions will be available. Translation of the SF-36 into German and French was done according to the Translation Protocols of the International Quality of Life Assessment Group [39,40]. The SF-36 Health Survey and the HADS-D have been used in a range of intensive care-related studies [5,41,42]. An overview of all measurements is summarized in Tables 1 and 2.

**Table 1:** Measurements during ICU stay

Variables	Instruments	Sources
Pain (intensity, frequency)	Behavioural scale	PDMS/Q

Agitation (intensity, frequency)	RASS, CAM-ICU	PDMS/Q

Reasons for ICU admission (cause)	APACHE: diagnosis at admission	MR/Q

Severity of illness	APACHE II, SAPS II	PDMS

Note: RASS = Richmond Agitation-Sedation Scale. CAM-ICU = Confusion Assessment Method for the ICU. APACHE = Acute Physiology and Chronic Health Evaluation. SAPS = Simplified Acute Physiology Score. PDMS = Patient Data Management System. MR = Medical record. Q = Questionnaire.

**Table 2:** Measurements in the ICU group and the comparison group (Follow-up)

Variables	Instruments	Time of Measurement		
		**Baseline/1 week after discharge ICU**	**6 months**	**12 months**

Pain (intensity, frequency)	NRS	Q	Q	Q

Anxiety (intensity, frequency)	NRS, HADS-D	Q	Q	Q

Agitation (intensity, frequency)	CAM	Q	Q	Q

Mortality	Survival: Yes/No	Q	Q	Q

Morbidity	International classification of diseases, version 10	MR/Q	MR/Q	MR/Q

Quality of life (HR-QOL)^1^	SF-36	Q	Q	Q

Note: NRS = Numeric rating scale. HADS-D = Hospital Anxiety and Depression Scale. CAM = Confusion Assessment Method. SF-36 = Short Form 36 Health Survey. HR-QOL = Health-related quality of life. MR = Medical record. Q = Questionnaire.

### Procedure

Background data will be gathered via medical records and questionnaires. Medical and nursing staff will gather daily routine data regarding pain, anxiety, and agitation, along with any other information relevant to usual care in the ICU. Collection of data concerning pain, anxiety and agitation will be carried out by the staff who regularly care for the patients. For this study these caregivers have received special training. Data about the outcome indicators will be collected using questionnaires 1 week, 6 months and 12 months after discharge. It should be emphasized that participants in the ICU group will be asked one week after discharge to make an assessment of both their current HR-QOL and their HR-QOL, pain, anxiety and agitation intensity, before admission to the ICU. One week after ICU discharge, patients in the ICU group will be contacted and interviewed. The interviews will be carried out only if the patients are physically and psychologically fit to be interviewed. If necessary, help will be provided in filling out the questionnaire. All data will be collected using face-to-face (baseline) and telephone (follow-up) interviews and will be collected by 4 trained interviewers. The baseline interviews with all critically ill patients will take place in the hospital. All other interviews for both groups will take place by telephone. The SF36 and HADS-D will be mailed to all participants. In order for them to prepare themselves for the interview, both groups will be informed of the general nature of the questions ahead of time. Because unanswered questions would affect the evaluation of the questionnaires, an interviewer will check to ensure that all questions have been answered, and follow up in cases where information is lacking. If questionnaires are not returned, reminders will be sent to the participants.

### Ethical Considerations

The study protocol was approved by the Cantonal Ethics Commission of Bern. For the patient interviews during the first week (5 to 10 days) after the ICU stay, patients will be contacted, comprehensively informed about the study--verbally and in writing--and invited to participate. The participants in the comparison group will receive the same information. Study participants agree to participate via a written informed consent form. To ensure confidentiality, all data will be coded, and all personal data will be documented, archived, and analyzed anonymously, making it impossible to determine the identity of the individual participants.

### Data Analysis

The statistical analysis will be performed using the R Project for Statistical Computing. Descriptive statistical analyses (mean, median, interquartile range) will be used to describe and characterize the data. To indicate the strength and direction of relationships between variables, Spearman's rank correlation coefficient for ordinal data and Pearson's product-moment coefficient will be conducted, depending on measurement levels and data dispersion. The Mann-Whitney U Test will be used for continuous data when comparing groups, and random effects models will be used for the longitudinal data. Attrition (i.e., missing data) during the follow-up will be reported.

## Discussion

This article presents the design of a prospective longitudinal study investigating the associations between admission to the ICU and pain, anxiety, agitation, health-related quality of life, and use of the health care system in later life. Previous longitudinal studies have examined ICU patients' health-related quality of life [4,7-9] or traumatic memories of postoperative treatment [2,5]. The present study specifically assesses situations involving pain, anxiety and agitation in older patients, as well as their consequences. These symptoms can influence the long-term outcomes of older critically ill patients. The study will be conducted over a period of two years. Data on health-related quality of life are time dependent and related to the life situation of the participant. Changes that take place during the follow-ups, such as modifications in therapy or accidents, were integrated into the analysis, based on the various measurement techniques. The instruments used for the study represent a limitation.

Various studies have attempted to explain ICU stay-related variables by using identical generic instruments to compare critically ill patients' health-related quality of life with that of comparison groups from the general population [4,9,41,42].

Apparently, illness-specific influences on health-related quality of life are not identified by these generic instruments. Various studies assessed HR-QOL with a variety of instruments [19,43] In addition, the effort required for older people to fill out multiple questionnaires is not negligible. Some studies on critically ill patients have questioned the patients' families regarding their health-related quality of life [44,45]. However, due to the subjective nature of quality of life, the current study uses only interview and questionnaire data provided by the patients themselves, in order to more accurately describe the patients' own perception of their ICU stay. Until now, little research has focused on the short- and long-term consequences of pain, anxiety, and agitation in older patients, including the effect on the patients' use of the health care system. Because inadequate pain assessment and management may needlessly increase hospital readmissions and the use of the health care system, this topic should be of broad interest. This study will provide the first data on the situation in Switzerland.

## Competing interests

The authors declare that they have no competing interests.

## Authors' contributions

All authors contributed to the preparation of the manuscript, and participated in the research. All authors read and approved the final manuscript.

## Pre-publication history

The pre-publication history for this paper can be accessed here:

http://www.biomedcentral.com/1471-2318/11/52/prepub
